# Transcriptomic analyses of juvenile Striped Bass (*Morone saxatilis*) exposed to chronic and acute temperature change

**DOI:** 10.1371/journal.pone.0289372

**Published:** 2023-10-19

**Authors:** Faith M. Penny, Scott A. Pavey

**Affiliations:** Department of Biological Sciences and Canadian Rivers Institute (CRI Genomics), University of New Brunswick, Saint John, New Brunswick, Canada; National Cheng Kung University, TAIWAN

## Abstract

Striped Bass are economically important, migratory fishes, which occur across a wide range of latitudes. Given their wide-ranging nature, Striped Bass can cope with a broad range of environmental temperatures, yet the mechanisms underlying this ability have not been thoroughly described. Heat shock proteins (HSPs) are inducible molecular chaperones, which help mitigate protein damage resulting from increased temperatures. The importance of HSPs has been demonstrated in a number of fish species, but their role in Striped Bass is poorly understood. This study characterizes changes in gene expression in juvenile Striped Bass, following acute and chronic temperature change. Fish were acclimated to one of three temperatures (15, 25 or 30°C) and sampled at one of two treatments (control or after CT_max_), following which we assessed differential gene expression and gene ontology in muscle. It is clear from our differential expression analyses that acclimation to warm temperatures elicits more robust changes to gene expression, compared to acute temperature increases. Our differential expression analyses also revealed induction of many different heat shock proteins, including *hsp70*, *hsp90*, *hsp40* and other small HSPs, after both acute and chronic temperature increase in white muscle. Furthermore, the most consistent gene ontology pattern that emerged following both acclimation and CT_max_ was upregulation of transcripts involved in “protein folding”, which also include heat shock proteins. Gene ontology analyses also suggest changes to other processes after acclimation, including decreased growth pathways and changes to DNA methylation. Overall, these data suggest that HSPs likely play a major role in the Striped Bass’s ability to tolerate warm waters.

## Introduction

Temperature is often referred to as the “master abiotic factor” [1]–especially in poikilothermic animals, including most fishes–as its effects span nearly all levels of biological organization, from protein folding, through to behaviours and species distribution [2]. The capacity of fishes to acclimate to warmer temperatures not only allows a species to inhabit a wider range of environments, but is also likely to be beneficial during subsequent extreme temperature events [3]. On acute time scales, rapid tests, such as the critical thermal maximum (CT_max_) test, provide a useful tool to facilitate comparison of thermal tolerance among experimental groups [4, 5]. Wide ranging, migratory species routinely encounter changes in environmental temperature, on both the short-term (i.e. tidal) and long-term scale (i.e. seasonal). This makes such fishes an useful candidate to explore the breadth of chronic and acute temperature stress [6].

The Striped Bass, *Morone saxatilis*, are a wide-ranging, anadromous fish, with important recreational and commercial value [7]. Striped Bass naturally inhabit many coastal rivers along the eastern seaboard of the USA and Canada and migrates upstream to spawn [7]. While the commercial harvest of Striped Bass has mostly reopened following a stock crash in the 1980’s, it has also become the most-sought after saltwater fish in the USA by recreational anglers, with a recreational harvest of 15.8 million tones in 2021 [7, 8]. In the northern stretches of its range, Striped Bass have come under scrutiny as a putative predator of Atlantic salmon smolts, especially in areas which Atlantic salmon listed as endangered, such as the Inner Bay of Fundy [9]. Penny and Pavey [10] showed that even Northern Striped Bass have a large capacity to acclimate to very warm waters for many weeks (i.e., 4 weeks at 30°C) with no mortality and little change in hematology, in contrast to the cold-water Atlantic Salmon. This impressive ability to cope with warm temperatures may be beneficial in warming waters, yet the underlying mechanisms that drive this ability are yet to be determined.

One of the most ubiquitous cellular mechanisms animals use to deal with heat stress are the heat shock proteins. Heat shock proteins (HSPs) are intracellular proteins found in nearly all organisms. HSPs are generally grouped by their molecular weights, with the most commonly studied groups being HSP70 (68–73 kDa) and HSP90 (85–90 kDa), though both smaller and larger HSPs also exist [11, 12]. While the structure of HSPs vary, these proteins generally act as molecular chaperones to help mitigate protein damage, misfolding and coagulation caused by stressors, including heat stress [2, 11, 13]. The induction of HSPs following a stress is commonly referred to as the Heat Shock Response (HSR). In fishes, the HSR is commonly includes *hsp70* and *hsp90*, although the extent and diversity of this response varies among fish species, and even populations within species [11, 12, 14, 15]. Within the HSP family, some are expressed constitutively (e.g. *hsc70*, the constitutive isoform of *hsp70*), while others can be rapidly induced by stressors (e.g., *hsp70*). This ability of a fish to induce an HSR is also likely to be an important deciding factor to whether the fish can acclimate to different temperatures [11]. Owing to the cosmopolitan nature of HSPs, it is likely that they play an important role in allowing Striped Bass to withstand temperature changes. Geist, Werner [16] detected induction of *hsp70* but not *hsp90* during exposure to toxic metals in Striped Bass kidney, yet outside of this, the HSR of these fish has not been described. The regulation of HSPs is believed to be controlled at the transcriptional level, with the resultant translation to useable HSPs swiftly following [11], thus measuring HSP-related RNA levels are a good surrogate for the relevant proteins levels. As such, high-throughput sequencing technology offers an opportunity to profile many different potentially induced HSPs, without *a priori* knowledge of the HSR.

The objective of this study is to describe the transcriptional changes elicited by chronic and acute heat stress in northern Striped Bass, using tissues collected by Penny and Pavey [10]. The previously published physiological data demonstrates an impressive ability of these fish to withstand chronic high temperatures, thus we aim to elucidate the underlying mechanisms. Given the ubiquitous and important role of HSPs in other species of eurythermal fishes, we also aim to describe the HSR in Striped Bass. We hypothesize that HSPs will play an important role in the HSR of Striped Bass.

## Materials and methods

### General husbandry

Juvenile Striped Bass (mass = 74.5 ± 3.4 g; fork length = 19 ± 0.4 cm; mean ± standard error) were raised from wild-caught eggs, spawned naturally in the Shubenacadie River (Nova Scotia, Canada). Experimental husbandry and sampling was performed at the University of New Brunswick’s Saint John campus. Prior to experiments, all fish were maintained in circular, fiberglass tanks (~750 L) with a constant flow of new, fresh (0 ‰), dechlorinated municipal water on a 14:10 hour light:dark rotation. Fish were fed daily to satiation (commercial Trout pellets; 3mm Vita Pellets; EWOS) but fasted for 24 hour prior to all experimental procedures or sampling (i.e. before CT_max_ trails or acclimation sampling). Before experimental procedures, temperatures were maintained at 15°C using multiple 800-watt submersible heaters (Aquatop Titanium 800 W; TH800W) with external temperature controllers (Inkbird; ITC-308).

### Experimental design

The overarching experimental design included acclimation of Striped Bass to one three temperatures (15, 25 or 30°C) and sampled at one of two treatments (control or at CT_max_). For each of these acclimation temperature x treatment combinations, seven fish were sampled (N = 7 x 3 temperatures x 2 treatments = 42 individual fish total). Acclimation to experimental temperatures was accomplished in the previously described husbandry tanks, with three replicate tanks for each temperature. Temperatures were increased by 1 to 1.5°C per day until the appropriate acclimation temperature was reached, following which the fish were maintained at this temperature for at least four weeks before further manipulation.

Following the four week acclimation period, two or three fish from each of the three replication temperature tanks were haphazardly selected for CT_max_ trials (N = 2–3 fish x 3 tanks/temperature = 7 total per acclimation temperature). Another seven fish were randomly selected in the same manner and sampled without further temperature manipulations (i.e. control fish). For simplicity, we henceforth refer to fish sampled as the acclimation controls (i.e., acclimated but no CT_max_) as the “acclimation” groups and those after CT_max_ simply as the “CT_max_” groups. Prior to commencing the CT_max_ trial, the selected fish was placed in the experimental chamber, at its respective acclimation temperature, and allowed to acclimatize for one hour. Further details of experimental chamber and temperature regulation can be found in [10]. Following the acclimatization period, the temperature of the experimental chamber was increased by 0.3°C · min^−1^ (18°C · hour^−1^) until the point of loss of equilibrium (LOE), at which time the temperature (CT_max_) was recorded and the trail stopped. LOE was defined as the point where the fish was no longer able to maintain a dorsal-ventral position.

### Tissue sampling

Immediately following LOE, each fish was euthanized via overdose in 100 mg · ml^−1^ of tricaine methanesulfonate (TMS; Cat: E10521; Sigma-Aldrich) buffered with sodium bicarbonate (200 mg · ml^−1^) followed by cervical dislocation. Hematological procedures and results are published by Penny and Pavey [10]. A sample of white epaxial muscle tissue was excised from the left trunk, from the dorsal end to the pectoral fin. All skin and non-white muscle was removed and then the muscle was immediately placed in 5mL of RNA storage solution (combined solution of 3.5 M ammonium sulfate, 0.05 M EDTA and 0.1 M sodium citrate at pH 5.2), stored at 2°C for 24 hours, and then stored at -80°C until RNA extraction.

### RNA extraction, DNA removal and pre-library preparation quality control

All RNA extraction, purification and library preparation was completed at the University of New Brunswick Saint John. Each white muscle sample was thawed on ice and a ~100mg section of tissue was dissected. The excess storage solution was blotted off, and then the tissue was placed in 1000 μL of TRIzol reagent (Cat:15596026; ThermoFisher; Waltham, USA) along with a 3mm tungsten ball. The samples were then homogenized using an oscillating mixer mill (Retch MM400) for 3 minutes at 25.0 Hz. Total RNA was then extracted from the homogenized tissues using PureLink RNA Mini Kit (Cat: 12183020; ThermoFisher; Waltham, USA) according to the manufacturer’s directions aside from the following changes: elution was preformed twice, with 30 μL of RNAse-free water heated to 37°C; prior to the first elution; 0.5 μL of recombinant ribonuclease inhibitor (RNaseOUT; Cat: 10777019; ThermoFisher; Waltham, USA) was added to the recovery tubes prior to the first elution.

Following extraction, quality and concentration of each sample was determined using microvolume spectrophotometry (NanoDrop 2000; ThermoFisher) and then normalized to ~200 ng/μL. Each sample was then DNase treated (Turbo DNA-Free Kit; Cat: AM1907; Invitrogen; Waltham, USA). The quality of each sample was then assessed with chip-based capillary electrophoresis (RNA 6000 Nano Kit; Agilent Bioanalzyer 2100; Agilent; Santa Clara, USA) and then quantified using fluorescence-based RNA-quantification assay (Quant-it™ RiboGreen RNA Assay Kit; Cat: LSR11490; Invitrogen; Waltham, USA). The concentration of all samples were normalized to 10 ng/μL for library preparation.

### RNA library preparation and sequencing

Two mRNA sequencing lanes were created, each with 21 multiplexed individual fish samples. To help mitigate sequencing lane effects, samples were randomized across each lane, but still ensuring that roughly half of the replicates from each temperature x treatment groups were on each lane. The libraries were prepared using the following pre-prepared kits, following the manufacturer’s directions: magnetic bead isolation (NEBNext Poly(A) mRNA Magnetic Isolation Module (Cat: E7490; New England Biolabs; Ipswich, USA); NEBNext Ultra II Directional RNA Library Prep Kit for Illumina (Cat: E7760; New England Biolabs; Ipswich, USA); NEBNext Multiplex Oligos for Illumina (Cat: E7335 & E7500; New England Biolabs; Ipswich, USA). Creation of cDNA from isolated RNA is included in the NEBNext protocol. Prior to final normalization and pooling, individual library quality was assessed using chip-based capillary electrophoresis (High Sensitivity DNA Kit; Agilent Bioanalzyer 2100) and then quantified using qPCR (NEBNext Library Quant Kit for Illumina, Cat: E7630; New England Biolabs; Ipswich, USA). All library preparation was completed with the aid of an automated liquid handling system (epMotion 5075t; Cat. 5075006022; Eppendorf; Hamburg, Germany). The two resultant multiplexed libraries were sequenced on the Illumina HiSeq 4000 platform (paired-end; 150BP), performed by GENEWIZ (New Jersey, USA).

### Quality control and alignment

Following demultiplexing by GENEWIZ, all of the samples were trimmed with Trimmomatic 0.39 [17]. Trimming was done based on residual adapters and barcodes as well as quality settings as follows: minimum read length = 35; sliding window = 4:20; ILLUMACLIP: 2:30:10. Pre and post trimming quality was visually assess using FastQC [18].

The Striped Bass reference genome (BioProjects Accession #: PRJNA266827; NCBI Project#: 266827) was used for alignment. The reference genome indexing and alignment were preformed using HISAT2 version 2.2.1.0 [19]. The resultant.sam files were converted to.bam and indexed using StringTie 1.3.5 [20]. Summary tables of raw read counts were compiled using htseq-counts 0.12.4 [21].

### Differential expression analyses

Differential expression analyses was performed using DESeq2 [22]. Experimental design settings for the DESeq2 object were defined by temperature, treatment and sequencing lane. Differential expression was examined via two methods: First, the effects of acclimation temperature were explored by comparing the 25°C and 30°C acclimation control fish each to the control fish acclimated to 15°C; Secondly, differential expression in response to CT_max_ was calculated by comparing fish at CT_max_ to the acclimation control fish at the respective temperature. These summary lists of relevant temperature x time combinations were extracted using the DESeq2::results() function. DEGs were categorized as up or down regulated by whether the resultant fold-changes were greater than (up) or less than (down) 1. Significant differentially expressed genes (DEGs) were selected using the Benjamini-Hochberg adjusted p-values with a cut off of 0.05. After rlog transforming the relative expression values, principal component analyses was performed on all of the expressed genes to visualize the effects of treatment, temperature and sequencing lanes. Heat plots of DEG groups of interest were also created using fold change (log2 or raw as appropriate) as the input value. All differential expression and figure creation were performed using R base plotting functions [23], aside from the Venn diagrams, which were created with eulerR [24].

### Gene ontology and clustering

Gene ontology (GO) analyses were performed on the DE gene lists using DAVID’s functional annotation tools [25], and the *Danio rerio* identifier. GO term lists were created from both up and downregulated DEG lists separately. Resultant GO terms were further refined selected from the “FAT” lists using the following criteria, according to Huang, Sherman [26]: Benjamini-Hochberg adjusted P-value < 0.05; fold enrichment ≥ 1.5; minimum number of DEGs/term = 5. Due to lower significant GO term numbers in the other categories, only GO terms from the “biological processes” category were used in clustering analyses. Where the resultant GO biological processes term list was greater than ten, semantic clustering analysis (REVIGO) was used to further reduce the GO list and examine the higher-level patterns [27]. Clustering analysis results were used to select representative GO terms for visualization in bubble plots (VisGO ref) in order to allow comparison of fold-enrichment, gene count and adjusted p-values across temperature and treatment groups.

## Results

### Summary of sequencing and alignment

The average number of reads/sample was similar across both sequencing lanes, with more than 21 million reads/ sample overall and 918 million reads total across both sequencing lanes (Table 1). After trimming, roughly 91% of raw reads remained. Of the possible mappable genes in the reference genome, ~89% of the gene had non-zero normalized read counts. The proportion of aligned annotated genes was similar to the proportion annotated in the reference genome (Table 1).

**Table 1:** Summary of sequencing results from juvenile Striped Bass exposed to acute (CT_max_) and chronic (acclimation) temperature change.

Metric	Reads		
**Total reads**			
Lane 1	452,810,110		
Lane 2	465,568,797		
	918,378,907		
**Reads/ Sample (mean ± SE)**			
Lane 1	21,562,386	±	707,609
Lane 2	22,169,943	±	1,566,307
OVERALL	21,866,164	±	850,144
OVERALL after Trimming	20,381,742	±	673,475
Percent reads removed by trimming	9.1	±	0.3%
Alignment Success/ Sample	72.3	±	0.4%
	*All*	* *	*Annotated*
# of Mappable Genes in Reference Genome	22,733		14,217 (63%)
# of Genes with > 0 Normalized Counts	20,195		13,052 (65%)

### Patterns of differentially expressed genes across treatments

**Fig 1:**
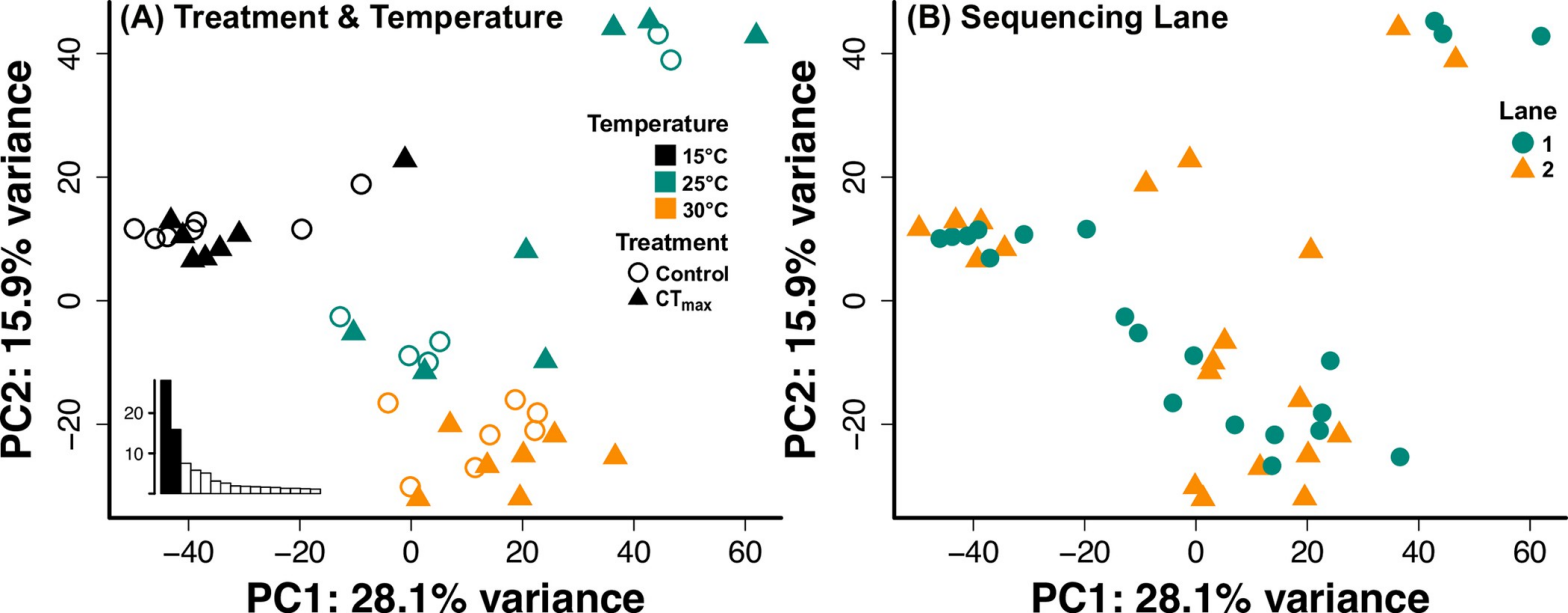
Principal components analysis of gene expression from juvenile Striped Bass exposed to one of two treatments (control or CT_max_) and acclimated to one of three temperatures (15, 25, or 30°C). Plot points are formatted to highlight the patterns of treatment & temperature (A) or sequencing lane (B). Plot (A) inlay is the scree plot created from the PCA analysis.

Principal components analyses suggested that the effect of acclimation temperature had a greater effect on expression than did subsequent temperature increases to CT_max_ (Fig 1A). Replicate fish grouped roughly by acclimation temperature, mostly spread along PC2 regardless of CTmax or sham treatment. Furthermore, fish acclimated to 15°C were grouped towards the lower values of PC1 while 25°C and 30°C fish mostly overlap along PC1. Unlike acclimation temperature, there is no obvious distinction between control and CT_max_ fish within their acclimation temperature “cloud”. When sequencing lane information is assessed by PCA, there is significant overlap and no distinct grouping of replicates based on sequencing lane (Fig 1B).

**Fig 2:**
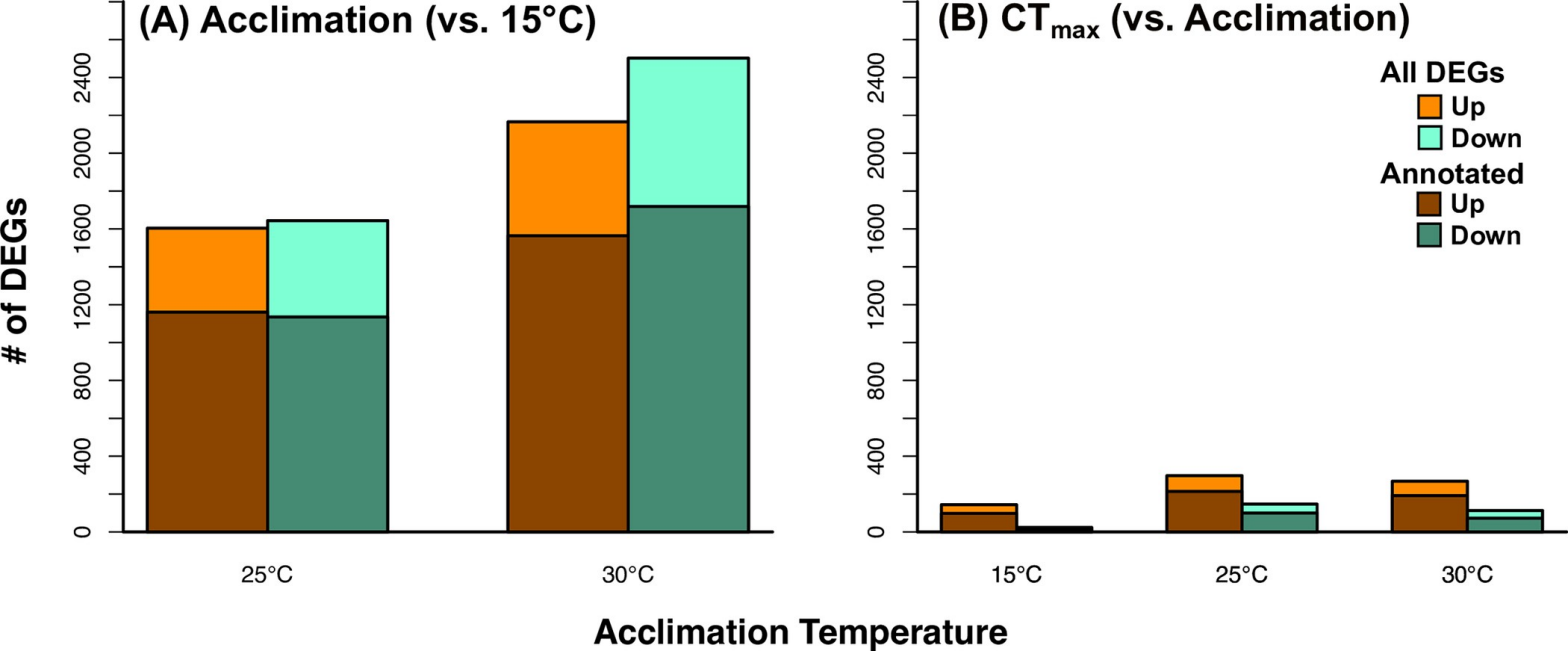
Differentially expressed genes (DEGs) from juvenile Striped Bass, acclimated to one of three temperatures, then sampled as acclimation controls or following a CT_max_ trial. For acclimation (A), differential expression is defined as difference from 15°C acclimated fish; For CT_max_ (B), differential expression is defined as difference from respective temperature acclimation control. DEGs are summarized both as total count, subtotal of annotated genes, and direction of change. Up = increased expression at CT_max_ vs control; Down = decreased expression at CT_max_ vs control.

The summary of DEG counts also showed a much larger effect of acclimation compared to that of CT_max_ (Fig 2). This is suggested by the much higher numbers of significant DEGs found in the acclimation comparisons (Fig 2A) versus the nearly order of magnitude lower number found in CT_max_ comparisons (Fig 2B). The pattern of total DEGs also differs between acclimation and CT_max_. In acclimated fish, the 30°C treatment elicited the highest total DEGs, whereas the 25°C treatment had the largest number in CT_max_ comparisons. For both acclimation comparisons, the number of up vs down DEGs were similar (Fig 2A). Contrarily, the response in fish at CT_max_ was highly skewed toward upregulated genes, with roughly 2x more up regulated DEGs vs down regulated (Fig 2B).

**Fig 3:**
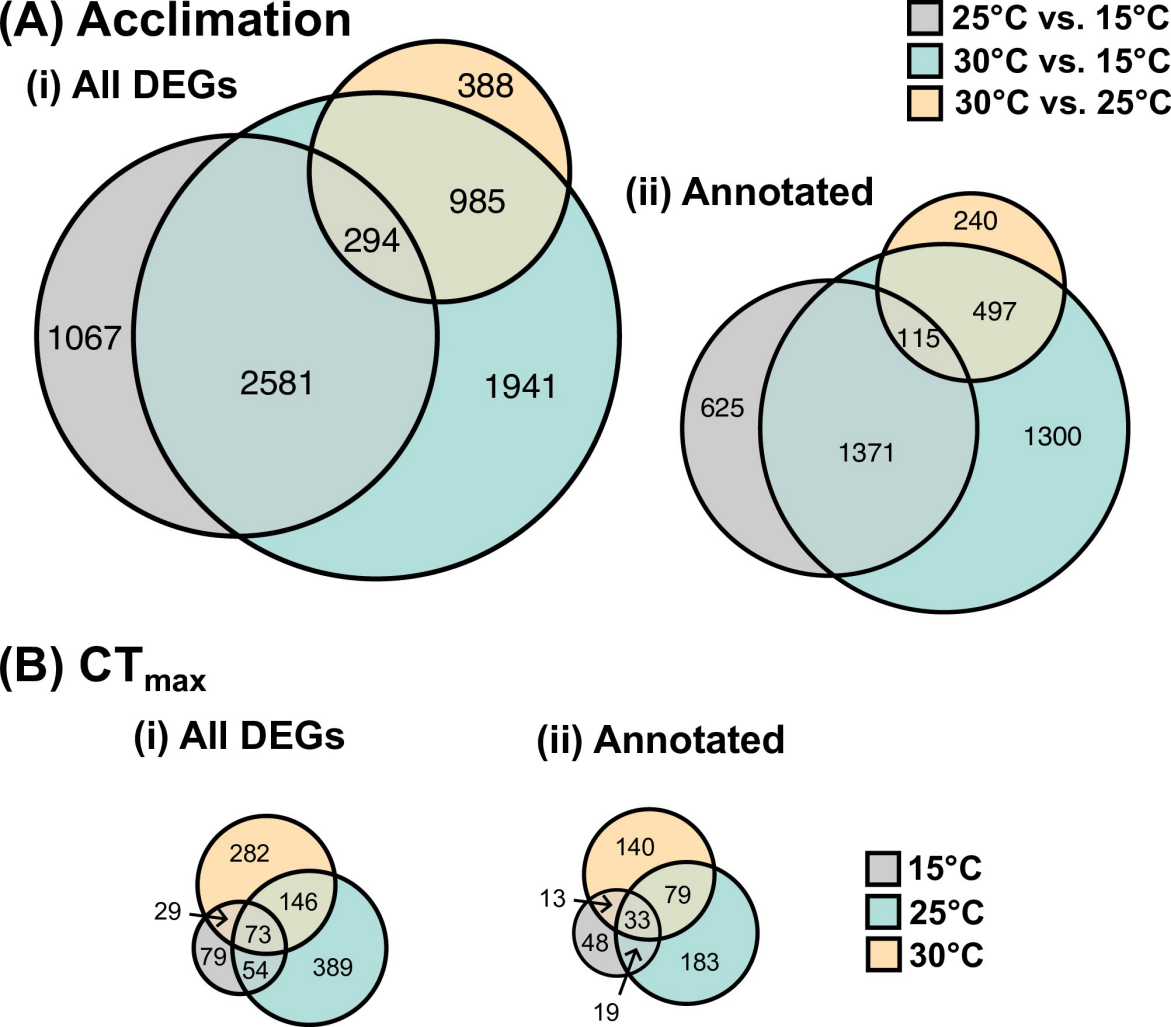
Euler diagrams of differentially expressed genes (DEGs) from juvenile Striped Bass from two treatments (A: Acclimation or B: CT_max_) and acclimated to one of three temperatures. DEGs are summarized both as total count (i) and whether the resultant genes are annotated (ii). The area of each circle is proportional to the respective number of DEGs, both within and among subsections of the figure (e.g. CTmax had fewer DEGs than Acclimation, and is thus proportionally smaller).

**Fig 4:**
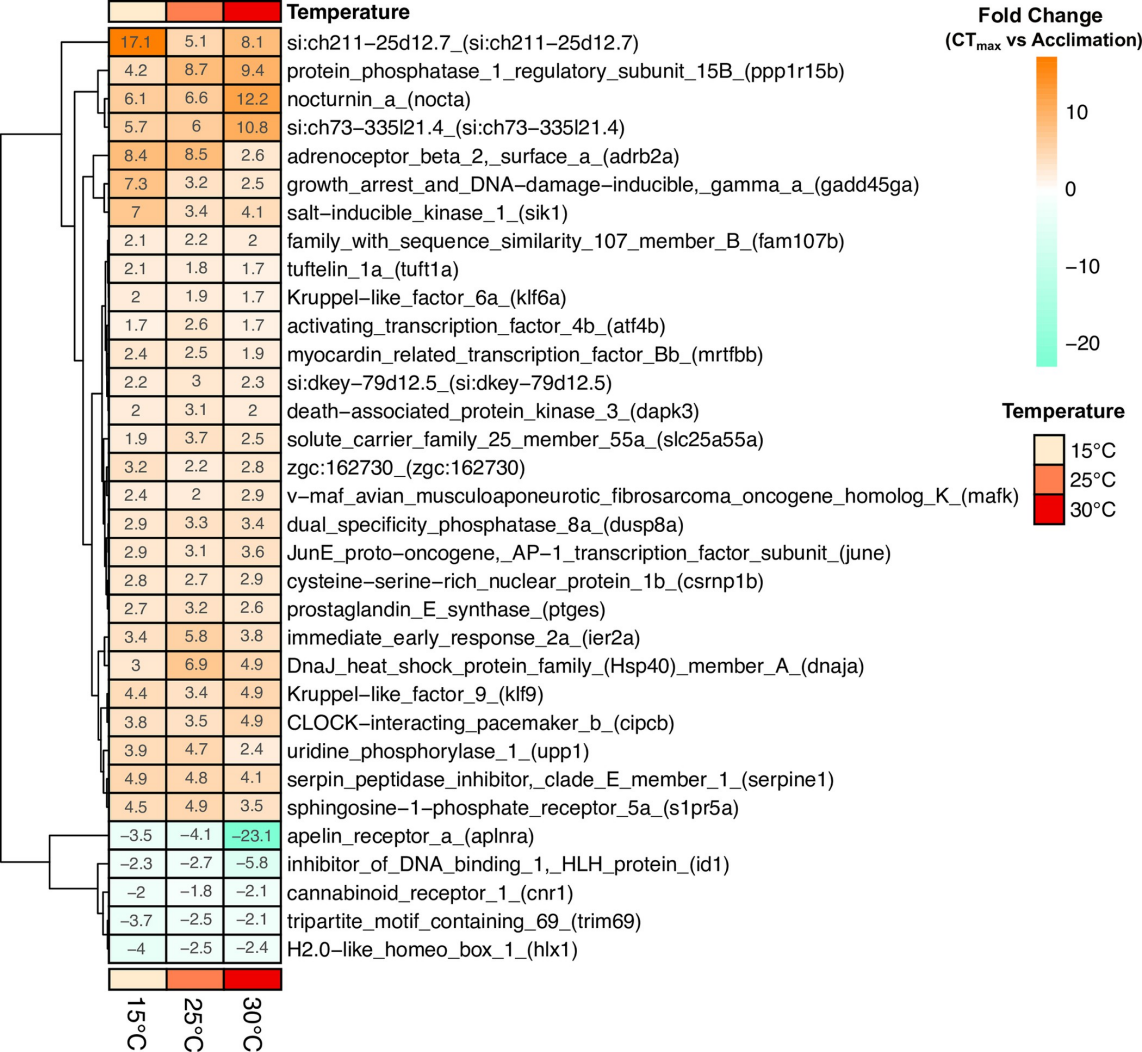
Heatmap of all significant annotated & differentially expressed genes at CT_max_ (vs acclimation control) shared across all three acclimation temperatures. Differential expression is defined as difference from respective temperature acclimation control. All values are displayed as actual fold change.

Within both acclimation and CT_max_ comparisons, each temperature elicited a private subset of genes, in addition to some shared genes (Fig 3). The largest number of private genes within acclimation were found at 30°C (Fig 3Ai), with a large proportion of these being annotated (Fig 3Aii). Fish acclimated to 25°C also had the largest number of private DEGs at CT_max_ while the smallest private DEG list at CT_max_ was at 15°C (Fig 3Bi). All three acclimation temperatures shared a subset of DEGs at CT_max_, 33 of which were annotated (Fig 3Bii). Of these shared annotated genes, the direction of change was the same across all acclimation temperatures, with the majority of genes being upregulated (Fig 4).

### Differential expression of heat shock proteins

**Fig 5:**
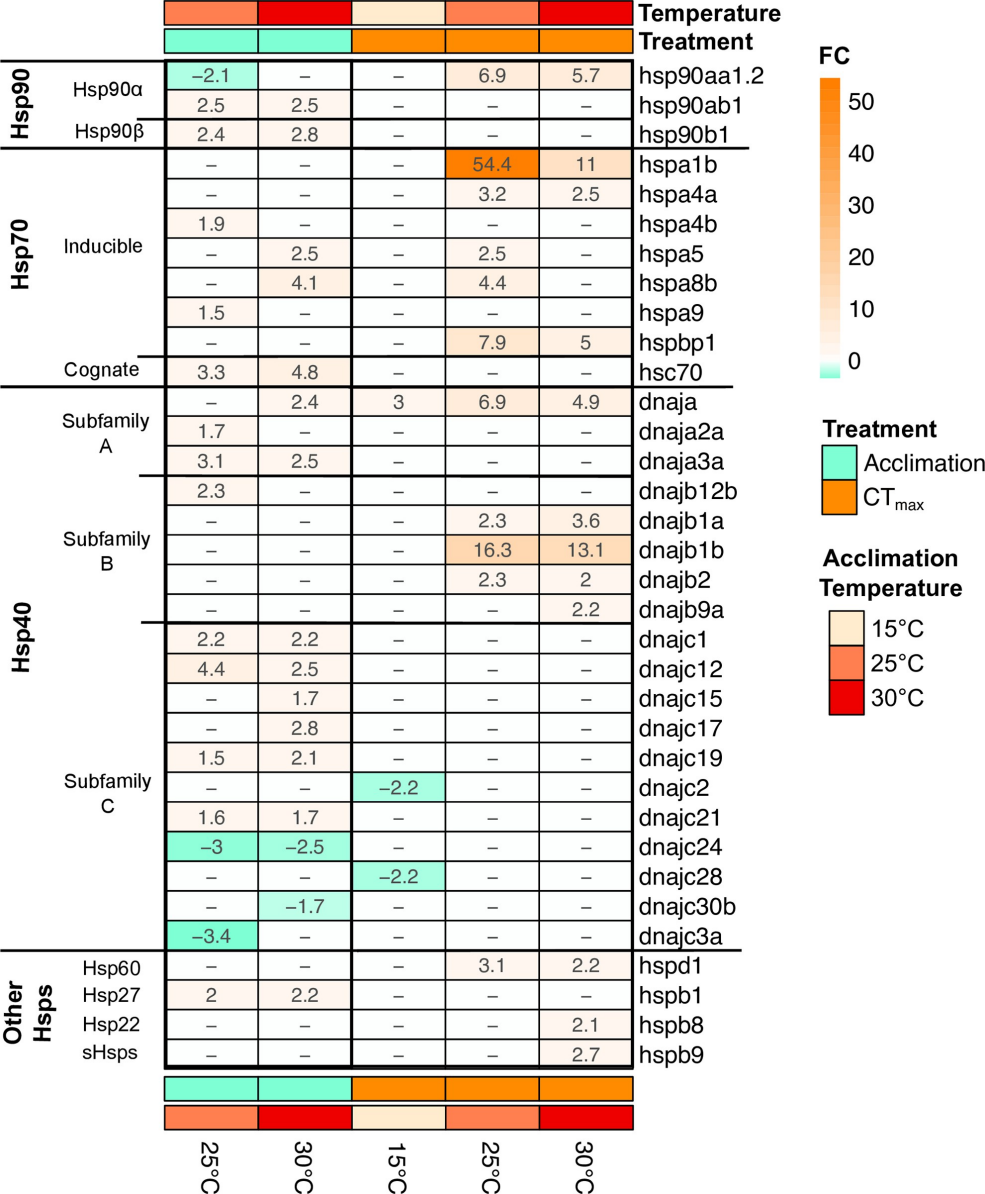
Heatmap of all significant annotated differentially expressed heat shock protein (Hsp) genes between acclimation temperature within two treatments (acclimation or CT_max_). For acclimation, differential expression is defined as difference from 15°C acclimated fish; For CT_max_, differential expression is defined as difference from respective temperature acclimation control. All values are displayed as actual fold change. sHsp–small family HSPs.

Thirty-four genes directly related to heat shock proteins were differentially regulated in at least one of the treatment groups (Fig 5). These DEGs relate to multiple HSP types, including hsp90, 70, 40 and some smaller HSPs. Generally, these HSP were upregulated at CT_max_ and warmer acclimation temperatures. The greatest variety and number of HSP-related genes were found in acclimation comparisons, with acclimation to both 25 and 30°C yielding a similar number of HSP DEGs, most of with are upregulated. The highest fold-change HSPs were found after CT_max_, specifically upregulation of subunits of inducible hsp70 (*hspa1b*) and hsp40 (*dnajb1b*). No HSP genes were upregulated at CT_max_ in 15°C acclimated fish; the only HSP changes at this temperature was two subunits of hsp60, which were down regulated.

### Gene ontology

In nearly all comparisons, the “biological processes” GO category had by far the highest significant terms compared to the other GO categories (Table 2). Similar to the patterns seen in DEG lists and PCA, acclimation comparisons yielded more significant GO terms than did CT_max_. The highest number of significant GO terms were found in fish acclimated to 30°C. At CT_max_ the 25°C fish produced a greater number of DEGs (Figs 2B and 3B), the 30°C fish produced a larger number of significant GO terms. The number of DEGs found at CT_max_ in 15°C were too short to allow for gene enrichment analyses.

**Table 2:** Counts of significant gene ontology (GO) terms resulting from differential expression analyses of juvenile Striped Bass acclimated to one of three temperatures (15, 25, or 25°C) after acclimation or CT_max_.

Group	Acclimation Temperature	Biological Processes			Molecular Functions			Cellular Components		
		Down	Up	Total	Down	Up	Total	Down	Up	Total
Acclimation	25°C	143	111	254	14	15	29	0	38	38
	30°C	278	137	415	34	14	48	51	0	51
CTmax	15°C	n/a	15	15	n/a	15	15	n/a	1	1
	25°C	n/a	21	21	n/a	3	3	n/a	1	1
	30°C	n/a	57	57	n/a	21	21	n/a	1	1

For acclimation, differential expression is defined as difference from 15°C acclimated fish; For CT_max_, differential expression was defined as difference from respective temperature acclimation control. Significant GO terms were defined as those which fold-enrichment > 1.5 and Benjamini-Hochberg p-value < 0.05. Counts are summarized by the three GO term categories (biological processes, molecular function or cellular components).

n/a = DEGs list was too short for GO analyses

**Fig 6:**
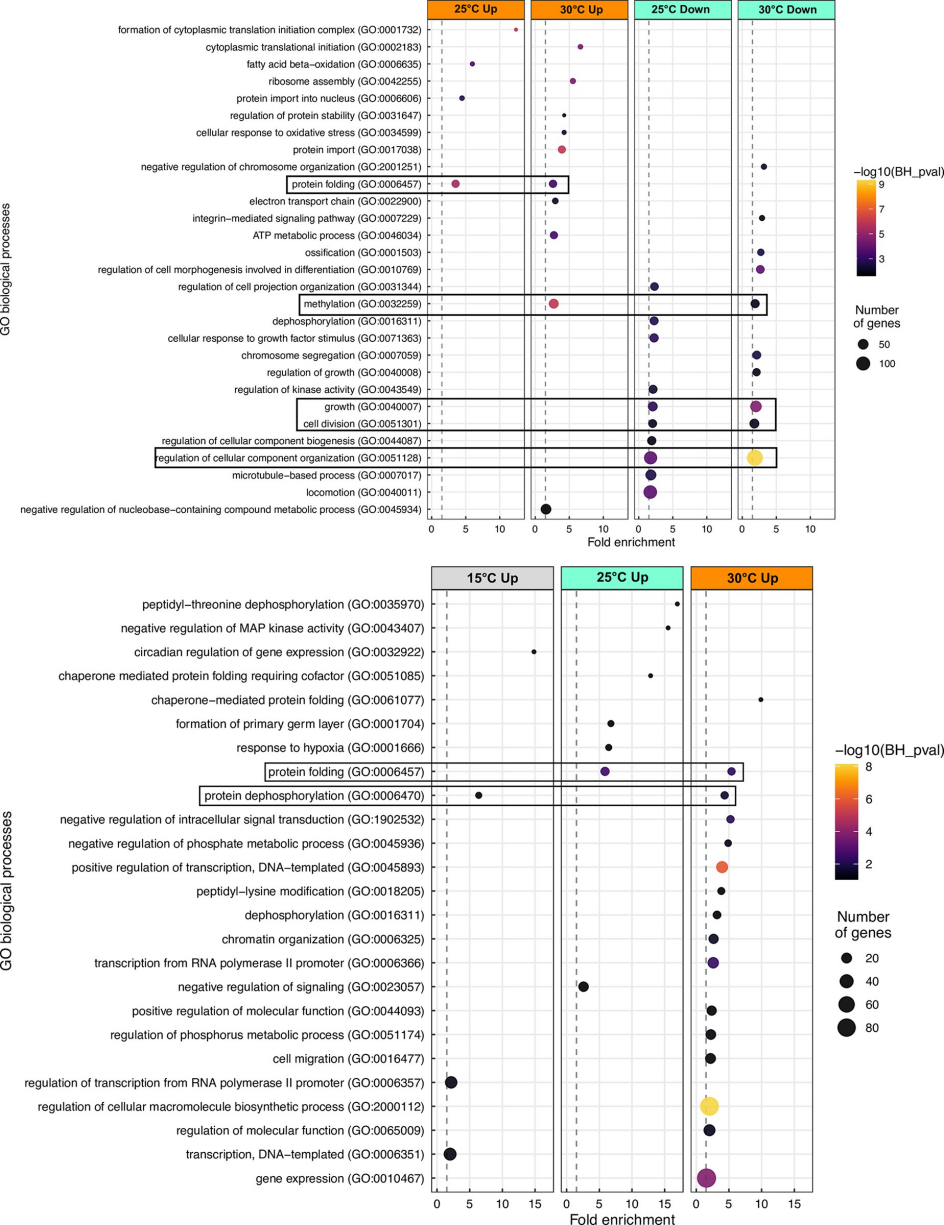
Bubble plots of the top 10 representative enriched GO terms (biological processes) in juvenile Striped Bass acclimated to various temperatures, and from one of two treatments (A = Acclimation; B = CT_max_). For acclimation, differential expression was defined as difference from 15°C acclimated fish; For CT_max_, differential expression was defined as difference from respective temperature acclimation control. Top representative GO terms were selected based on clustering analysis (REVIGO). GO terms are ordered top to bottom by decreasing fold-enrichment. Boxes highlight GO terms which are shared across at least two acclimation temperatures. The dotted line represents the lower fold-enrichment cut-off of 1.5. Only upregulated GO terms are displayed for CT_max_ data (B) the respective DEG lists were too small to allow for GO analysis.

**Fig 7:**
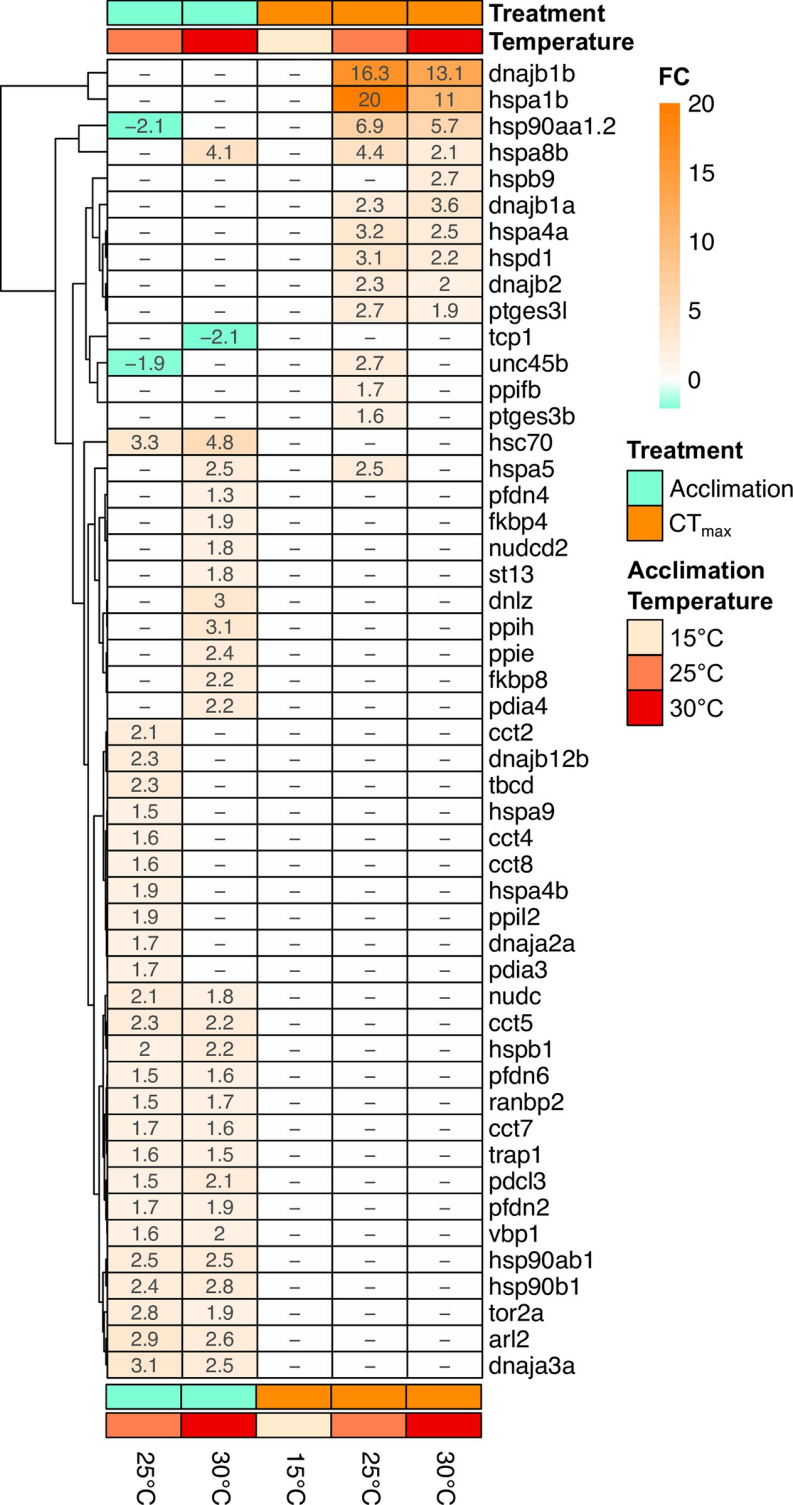
Heatmap of all significant annotated differentially expressed genes from the “protein folding” gene ontology term (GO:0006457) found in juvenile Striped Bass acclimated to various temperatures, and from one of two treatments (acclimation or CT_max_). For acclimation, differential expression was defined as difference from 15°C acclimated fish; For CTmax, differential expression was defined as difference from respective temperature acclimation control. All values are displayed as actual fold change.

**Fig 8:**
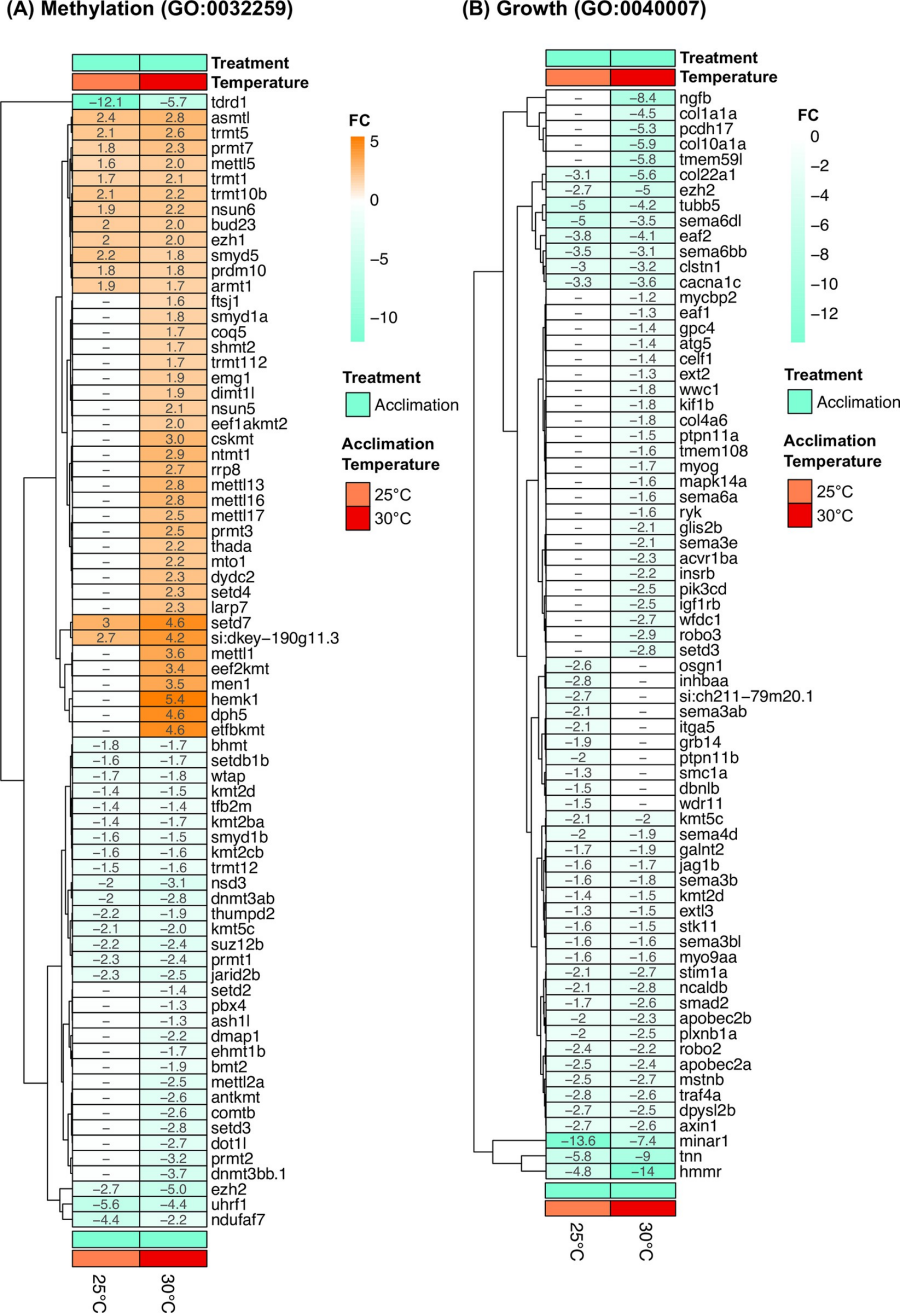
Heatmaps of all significant annotated differentially expressed genes from the gene ontology terms “Methylation (A; GO:0032259) or “Growth” (B; GO:0040007) found in juvenile Striped Bass acclimated to various temperatures, and from one of two treatments (Acclimation or CT_max_). For acclimation, differential expression was defined as difference from 15°C acclimated fish; For CT_max_, differential expression was defined as difference from respective temperature acclimation control. All values are displayed as actual fold change.

One GO term was found to be upregulated in multiple temperatures in both acclimation and CT_max_ comparisons: protein folding (Fig 6). Genes related to protein folding were significantly upregulated at 25 and 30°C following both acclimation (Fig 6A) and CT_max_ (Fig 6B), with higher fold enrichments found at CT_max_. While this term was upregulated following both acclimation and CT_max_, the specific genes upregulated appear to differ between these treatments (Fig 7). Nearly all of the genes upregulated at CT_max_ are HSP related genes. Comparatively, acclimation to increased temperatures resulted in a more varied list of protein folding genes, which includes some HSP genes (e.g. *hsc70*, *dnaka12b*) but chaperonins (*cct2*, *cct4*, *cct5*, *cct7* & *cct8*) (Fig 7). No other top GO terms were found in common at both acclimation and CT_max_. A greater variety of top GO terms were found to be shared across temperatures in acclimation compared to CT_max_. (Fig 6) For example, genes related to growth and cell division were downregulated following acclimation to higher temperatures (25 and 30°C) (Fig 8A) whereas methylation related genes showed a mixed response, in that multiple related genes were both up and down regulated at 30°C (Fig 8B).

## Discussion

Both chronic (4 week acclimation) and acute heat exposure (at CT_max_) initiated a heat shock protein transcriptomic response in juvenile Striped Bass. This is evident by the consistent upregulation of the ‘protein folding’ gene ontology and suites of HSP-related genes in both acute and chronic heat exposures. While heat shock proteins are found in nearly all organisms, the extent, type and patterns of HSP induction vary by organism, individual history, environment and many other factors [2, 12–15]. Of the many different HSPs, *hsp70* and *hsp90* are the HSPs are the most commonly described HSPs in fishes [12, 28]. Our data show that multiple genes related to these two HSPs were indeed upregulated following both acute and chronic heat stress in Striped Bass at 25°C and 30°C. Interestingly, fish acclimated to the control temperature of 15°C did not show an increase of HSPs or other protein folding genes at CT_max_, suggesting the HSR was not initiated. Logan and Somero (2010, 2011) detailed the differences between chronic (4 week) and acute heat stress on the HSR in *Gillichthys mirabilis* (longjaw mudsucker) and found that acclimation produced a much less robust transcriptomic response when compared to the acute response. This contrasts our findings in two ways: (1) the effects of acclimation was much greater than acute and (2) both acclimation and CT_max_ produced a strong HSP response. In addition to the upregulation of stress-inducible HSPs (i.e. *hsp90α* & *hsp70*), acclimation also elicited an upregulation of constitutive HSPs—*hsp90β* and *hsc70* [2]. We found that these constitutive HSPs to only be upregulated following acclimation whereas the stress-inducible HSPs are upregulated in all temperature treatments to some extent. Fangue, Hofmeister [15] documented higher levels of constitutive HSPs (*hsc70* and *hsp90β*) in warm-adapted Southern killifish (vs their Northern counterparts) and suggested this may lead to this population’s larger thermal tolerance range. Thus, our data suggests that Striped Bass’s multi-pronged HSP response is well adapted to cope with fluctuating temperatures. To my knowledge, no previous studies have examined temperature related induction of these common HSPs in Striped Bass, but Geist, Werner [16] reported induction of *hsp70* (but not *hsp90*; isoform not specified) following chronic (7-day) copper exposure. Furthermore, the *hsp70* induction reported by Geist, Werner [16] was found only in kidney tissue, and not white muscle, the tissue examined in the current study. Heat shock proteins have been relatively more studied in the related European Sea Bass (*Dicentrarchus labrax*), though much of this research used a targeted gene approach and focused on aquaculture practices. In *D*. *labrax*, significant induction of *hsp70* has been found following changes in diet [29, 30], handling stress [31], vaccination [32] and during larval development [33]. Cheng, Xia [34] used RNA-seq to assess transcriptomic changes in *D*. *labrax* after infection by a common pathogen, but found no induction of any HSP-related genes, while the pufferfish (*Takifugu rubripes*) also assessed in this study did show HSP induction. Our data suggests that the HSR (heat shock response) in Striped Bass do indeed launch a robust HSP response following both chronic and acute heat stress.

In addition to the commonly studied *hsp70* and *hsp90*, we also found upregulation of multiple genes related to *hsp40* in response to increased temperature. While many HSP-related research in fish biology focuses on *hsp70* and *hsp90* [11, 32, 35–37], it is not surprising to find induction of *hsp40* as well. *hsp40* is believed to work co-operatively with *hsp70*’s in protein folding, transport and degradation [38]. Specifically, *hsp40* acts by stimulating ATPase activity for the proper function of *hsp70* [39]. While hsp70 and hsp90 may be able to prevent protein aggregation during stress, the cooperation of hsp40 allows for actual renaturation of proteins [35]. We found that the treatments with the highest *hsp70*-related gene upregulation were also the treatments with the highest fold-change of *hsp40*. This co-upregulation was greatest at CT_max_ in the two highest acclimation temperature fishes, with lower fold-changes of these genes also differentially expressed at acclimation. In fishes, upregulation of *hsp40* has mainly been documented in response to exposure to infection agents, including in *Paralichthys olivaceus* (olive flounder), *Epinephelus coioides* (orange-spotted grouper), *Ictalurus punctatus* (channel catfish), and *Carassius auratus* (Crucian carp) [40–43]. The mixed direction response (e.g., some subunits upregulated and others downregulated) we found in *hsp40* subunit genes after acclimation is similar to that described in Channel Catfish liver following bacterial exposure [43]. Co-induction of *hsp40* with *hsp70* was described in longjaw mudsucker (*Gillichthys mirabilis*) following acute heat stress but not after acclimation to high temperature [37, 44]. This co-induction of *hsp40* and *hsp70* has not previously been described in Striped Bass or other member of this genus, to our knowledge.

Chronic temperature increases also appear to decrease processes related to growth. All of the DEGs found within the “growth” GO term were downregulated following acclimation to warmer temperatures (both 25 and 30°C vs 15°C), which suggests that growth is likely to be reduced overall. This trade-off would suggest that, while juvenile Striped Bass are able to survive at warmer temperatures (up to four weeks at 30°C), as demonstrated by Penny and Pavey (2021), it is likely not their thermal optimum. Decreases in whole-animal performance, including somatic growth, are described in Barton [45] as tell-tale signs of tertiary stress. This trade-off between warm temperature and decreased growth and has been described in many fishes [46–48]. Given that some function of HSPs are an active, ATP-dependent process (e.g. *hsp7*0 and 40 aided refolding [39]), the induction of the HSR seen in this study may further limit the energy reserves for somatic growth. Jutfelt, Norin [49] recently proposed that this reduced growth in warm conditions may be in an effort to protect aerobic scope, through reduced food consumption. The relationship of growth to temperature in Striped Bass has been well documented and is similar to other fishes, where growth tends to increase with temperature until the optimal temperature is surpassed [50–53]. So, while Striped Bass are capable of surviving at very warm (30°C) temperatures for many weeks, this downregulation of growth related genes suggests it is at the cost of fitness-related processes, like growth.

During chronic temperature increases, DNA methylation processes are also significantly modified. Unlike the clear transcriptional downregulation in growth related genes, methylation related genes are both up and down regulated, with the most DEGs found at 30°C vs. 15°C. As methylation may act to either increase or decrease expression, depending on the nature of the methylation, it is difficult to predict the exact outcome of the changes seen here. Nonetheless, it is likely that methylation is changing after acclimation to warmer temperatures. Changes in methylation has been documented in other fishes following chronic temperature change. For example, Beemelmanns, Ribas [54] found changes in CpG site methylation in Atlantic salmon exposed to chronic high temperature. Changes in methylation has also been implicated in facilitating phenotypic plasticity [55]. Epigenetic changes, such as methylation, can allow an organism to react to a changing environment by influencing the transcriptional response. Unlike evolutionary changes at the population-scale, such as allele frequency change, epigenetic processes may change the genetic ‘tools’ available to an individual many times within its own lifetime. Such epigenetic changes may have wide-ranging effects, as they may also be passed down to future generations [56]. Thus, the changes to methylation here likely serve as an indicator of the plasticity of Striped Bass to cope with new temperature regimes.

Along with changes related to protein folding, other genes were also more highly expressed at CT_max_ compared to sham treatments at all acclimation temperatures. One of these genes, *klf9* has also been found in two Sturgeon species at CT_max_ [57]. It has recently been suggested that Krüppel-like factor 9 (*klf9*), plays a role as a feedforward regulator of the transcriptional response to glucocorticoid stress pathway signaling [58]. This gene was upregulated at CT_max_ in all three acclimation temperatures in the current study, with a fold change similar to that previously published for juvenile Atlantic and Shortnose Sturgeons (FC = 3.4–4.9 in current study; FC = 3.6–3.7 in Sturgeons; Penny *et al*. 2022). Given the stressful nature of the CT_max_ trail procedures, the participation of the glucocorticoid pathway is not unexpected. Changes to Krüppel-like factor 6 (*klf6*), were also found in all CT_max_ treatments. In addition to their putative role in glucocorticoid processes, Krüppel-like factors are also zinc-finger transcription factors. Little other work has been done in fishes, though Yu, Li [59] characterized *klf9* in Grouper, and found significant upregulation following viral exposures.

Striped Bass are capable of existing in a wide range of temperatures, both under acute and chronic timelines, as demonstrated by their wide ranging, migratory nature. The data presented in this study suggests that changes to HSPs likely play a critical role in this tolerance, both in the chronic and acute time scale. An ability to both upregulate constitutive HSPs in white muscle and launch a robust acute heat shock response, provides protection from the protein damage common during heat stress. Furthermore, we have demonstrated *hsp40* mRNA is also induced following temperature increases, which likely acts to strengthen the HSP response in these fish as found in other species. Heat exposure also may decrease growth in long term and high temperature acclimation treatments reiterate that this is not the ideal temperature for Striped Bass. Nonetheless, these data show that Striped Bass are well-equipped to cope with large temperature changes on the short and longer-term timescale. This will undoubtedly serve them well in a warming environment.

## References

[1] Fry FEJ. Effects of the environment on animal activity. Toronto: University of Toronto Press; 1947.

[2] Hochachka PW, Somero GN. Biochemical adaptation: mechanism and process in physiological evolution. New York: Oxford University Press; 2002.

[3] Morley SA, Peck LS, Sunday JM, Heiser S, Bates AE. Physiological acclimation and persistence of ectothermic species under extreme heat events. Global Ecol Biogeogr. 2019;28(7):1018–37. doi: 10.1111/geb.12911

[4] Beitinger TL, Bennett WA, McCauley RW. Temperature tolerances of North American freshwater fishes exposed to dynamic changes in temperature. Environ Biol Fishes. 2000;58(3):237–75.

[5] Lutterschmidt WI, Hutchison VH. The critical thermal maximum: history and critique. Can J Zool/Rev Can Zool. 1997;75(10):1561–74. doi: 10.1139/z97-783

[6] Sunday JM, Bates AE, Dulvy NK. Global analysis of thermal tolerance and latitude in ectotherms. Proc R Soc Lond, Ser B: Biol Sci. 2011;278(1713):1823–30. doi: 10.1098/rspb.2010.1295 21106582 PMC3097822

[7] NOAA. 66th Northeast Regional Stock Assessment Workshop (66th SAW) Assessment Report. United States National Marine Fisheries, Service Northeast Fisheries Science, Center. 2019. 10.25923/nhqe-jd35

[8] NOAA. Species Directory: Atlantic Striped Bass 2022 [Available from: https://www.fisheries.noaa.gov/species/atlantic-striped-bass.

[9] Andrews SN, Hirtle SV, Linnansaari T, Curry RA. Consumption of Atlantic salmon smolt by striped bass: a review of the predator-prey encounter literature and implications for the design of effective sampling strategies. Fishes. 2019;4(4):50.

[10] Penny F, Pavey S. Increased acute thermal tolerance and little change to hematology following acclimation to warm water in juvenile Striped Bass, Morone saxatilis. Environ Biol Fishes. 2021;104(4):489–500.

[11] Basu N, Todgham A, Ackerman P, Bibeau M, Nakano K, Schulte P, et al. Heat shock protein genes and their functional significance in fish. Gene. 2002;295(2):173–83. doi: 10.1016/s0378-1119(02)00687-x 12354651

[12] Mohanty BP, Mahanty A, Mitra T, Parija SC, Mohanty S. Heat shock proteins in stress in teleosts. Regulation of heat shock protein responses: Springer; 2018. p. 71–94.

[13] Iwama GK, Thomas PT, Forsyth RB, Vijayan MM. Heat shock protein expression in fish. Rev Fish Biol Fish. 1998;8(1):35–56. doi: 10.1023/A:1008812500650

[14] Iwama GK, Afonso LO, Todgham A, Ackerman P, Nakano K. Are hsps suitable for indicating stressed states in fish? J Exp Biol. 2004;207(1):15–9. doi: 10.1242/jeb.00707 14638828

[15] Fangue NA, Hofmeister M, Schulte PM. Intraspecific variation in thermal tolerance and heat shock protein gene expression in common killifish, *Fundulus heteroclitus*. J Exp Biol. 2006;209(15):2859–72. doi: 10.1242/jeb.02260 16857869

[16] Geist J, Werner I, Eder KJ, Leutenegger CM. Comparisons of tissue-specific transcription of stress response genes with whole animal endpoints of adverse effect in striped bass (Morone saxatilis) following treatment with copper and esfenvalerate. Aquat Toxicol. 2007;85(1):28–39. doi: 10.1016/j.aquatox.2007.07.011 17767966

[17] Bolger AM, Lohse M, Usadel B. Trimmomatic: a flexible trimmer for Illumina sequence data. Bioinformatics. 2014;30(15):2114–20. doi: 10.1093/bioinformatics/btu170 24695404 PMC4103590

[18] Brown J, Pirrung M, McCue LA. FQC Dashboard: integrates FastQC results into a web-based, interactive, and extensible FASTQ quality control tool. Bioinformatics. 2017;33(19):3137–9. doi: 10.1093/bioinformatics/btx373 28605449 PMC5870778

[19] Wen G, editor A simple process of RNA-sequence analyses by Hisat2, Htseq and DESeq2. Proceedings of the 2017 International Conference on Biomedical Engineering and Bioinformatics; 2017.

[20] Pertea M, Pertea GM, Antonescu CM, Chang T-C, Mendell JT, Salzberg SL. StringTie enables improved reconstruction of a transcriptome from RNA-seq reads. Nat Biotechnol. 2015;33(3):290–5. doi: 10.1038/nbt.3122 25690850 PMC4643835

[21] Anders S, Pyl PT, Huber W. HTSeq—a Python framework to work with high-throughput sequencing data. Bioinformatics. 2015;31(2):166–9. doi: 10.1093/bioinformatics/btu638 25260700 PMC4287950

[22] Love M, Anders S, Huber W. Differential analysis of count data–the DESeq2 package. Genome Biol. 2014;15(550):10.1186. 10.1186/s13059-014-0550-8 PMC4302049 25516281

[23] R Core Team. R: A Language and Environment for Statistical Computing. Vienna, Austria: R Foundation for Statistical Computing; 2020.

[24] Larsson J. eulerr: area-proportional Euler diagrams with ellipses. 2018.

[25] DennisG, ShermanBT, HosackDA, YangJ, GaoW, LaneHC, et al. DAVID: database for annotation, visualization, and integrated discovery. Genome biology. 2003;4(9):1–11. 12734009

[26] Huang DW, Sherman BT, Lempicki RA. Systematic and integrative analysis of large gene lists using DAVID bioinformatics resources. Nature protocols. 2009;4(1):44–57. doi: 10.1038/nprot.2008.211 19131956

[27] Supek F, Bošnjak M, Škunca N, Šmuc T. REVIGO summarizes and visualizes long lists of gene ontology terms. PloS one. 2011;6(7):e21800. doi: 10.1371/journal.pone.0021800 21789182 PMC3138752

[28] Currie S, Schulte PM. Thermal Stress. In: Evans DH, Claiborne JB, Currie S, editors. The Physiology of Fishes, 4th Edition: CRC Press; 2013. p. 257–87.

[29] Montero D, Terova G, Rimoldi S, Betancor MB, Atalah E, Torrecillas S, et al. Modulation of the Expression of Components of the Stress Response by Dietary Arachidonic Acid in European Sea Bass (Dicentrarchus labrax) Larvae. Lipids. 2015;50(10):1029–41. doi: 10.1007/s11745-015-4057-1 26233819

[30] Bagni M, Romano N, Finoia MG, Abelli L, Scapigliati G, Tiscar PG, et al. Short- and long-term effects of a dietary yeast β-glucan (Macrogard) and alginic acid (Ergosan) preparation on immune response in sea bass (Dicentrarchus labrax). Fish and Shellfish Immunology. 2005;18(4):311–25. doi: 10.1016/j.fsi.2004.08.003 15561561

[31] Poltronieri C, Maccatrozzo L, Simontacchi C, Bertotto D, Funkenstein B, Patruno M, et al. Quantitative RT-PCR analysis and immunohistochemical localization of HSP70 in sea bass Dicentrarchus labrax exposed to transport stress. European Journal of Histochemistry. 2007;51(2):125–36. 17664163

[32] Mosca F, Romano N, Malatesta D, Ceccarelli G, Brunetti A, Bulfon C, et al. Heat shock protein 70 kDa (HSP70) increase in sea bass (Dicentrarchus labrax, L 1758) thymus after vaccination against Listonella anguillarum. Fish Physiol Biochem. 2013;39(3):615–26. doi: 10.1007/s10695-012-9724-z 23053607

[33] Bertotto D, Poltronieri C, Negrato E, Richard J, Pascoli F, Simontacchi C, et al. Whole body cortisol and expression of HSP70, IGF-I and MSTN in early development of sea bass subjected to heat shock. Gen Comp Endocrinol. 2011;174(1):44–50. doi: 10.1016/j.ygcen.2011.08.003 21872596

[34] Cheng JX, Xia YQ, Liu YF, Liu PF, Liu Y. Transcriptome analysis in Takifugu rubripes and Dicentrarchus labrax gills during Cryptocaryon irritans infection. J Fish Dis. 2021;44(3):249–62. doi: 10.1111/jfd.13318 33314157

[35] Somero GN. The cellular stress response and temperature: Function, regulation, and evolution. Journal of Experimental Zoology Part A: Ecological and Integrative Physiology. 2020;333(6):379–97. doi: 10.1002/jez.2344 31944627

[36] Roberts R, Agius C, Saliba C, Bossier P, Sung Y. Heat shock proteins (chaperones) in fish and shellfish and their potential role in relation to fish health: a review. J Fish Dis. 2010;33(10):789–801. doi: 10.1111/j.1365-2761.2010.01183.x 20678104

[37] Logan CA, Somero GN. Transcriptional responses to thermal acclimation in the eurythermal fish Gillichthys mirabilis (Cooper 1864). American Journal of Physiology-Regulatory, Integrative and Comparative Physiology. 2010;299(3):R843–R52. doi: 10.1152/ajpregu.00306.2010 20610827

[38] Li J, Qian X, Sha B. Heat shock protein 40: structural studies and their functional implications. Protein and peptide letters. 2009;16(6):606–12. doi: 10.2174/092986609788490159 19519518 PMC2755554

[39] Qiu X-B, Shao Y-M, Miao S, Wang L. The diversity of the DnaJ/Hsp40 family, the crucial partners for Hsp70 chaperones. Cellular and Molecular Life Sciences CMLS. 2006;63(22):2560–70. doi: 10.1007/s00018-006-6192-6 16952052 PMC11136209

[40] Dong C-W, Zhang Y-B, Zhang Q-Y, Gui J-F. Differential expression of three Paralichthys olivaceus Hsp40 genes in responses to virus infection and heat shock. Fish Shellfish Immunol. 2006;21(2):146–58. doi: 10.1016/j.fsi.2005.11.002 16377209

[41] Chen H-J, Li P-H, Yang Y, Xin X-H, Ou Y, Wei J-G, et al. Characterization and function analysis of Epinephelus coioides Hsp40 response to Vibrio alginolyticus and SGIV infection. Fish Shellfish Immunol. 2021;118:396–404. doi: 10.1016/j.fsi.2021.09.030 34571156

[42] Li X, Hu X, Lv A, Guan Z. Skin immune response to Aeromonas hydrophila infection in crucian carp Carassius auratus revealed by multi-omics analysis. Fish Shellfish Immunol. 2022;127:866–75. doi: 10.1016/j.fsi.2022.07.036 35850458

[43] Song L, Zhang J, Li C, Yao J, Jiang C, Li Y, et al. Genome-wide identification of Hsp40 genes in channel catfish and their regulated expression after bacterial infection. PLoS One. 2014;9(12):e115752. doi: 10.1371/journal.pone.0115752 25542027 PMC4277396

[44] Logan CA, Somero GN. Effects of thermal acclimation on transcriptional responses to acute heat stress in the eurythermal fish Gillichthys mirabilis (Cooper). American Journal of Physiology-Regulatory, Integrative and Comparative Physiology. 2011;300(6):R1373–R83. doi: 10.1152/ajpregu.00689.2010 21411771

[45] Barton BA. Stress in fishes: A diversity of responses with particular reference to changes in circulating corticosteroids. Integr Comp Biol. 2002;42(3):517–25. doi: 10.1093/icb/42.3.517 21708747

[46] Baldwin NS. Food Consumption and Growth of Brook Trout at Different Temperatures. Trans Am Fish Soc. 1957;86(1):323–8. doi: 10.1577/1548-8659(1956)86[323:FCAGOB]2.0.CO;2

[47] Schulte PM, Healy TM, Fangue NA. Thermal performance curves, phenotypic plasticity, and the time scales of temperature exposure. Integr Comp Biol. 2011;51(5):691–702. doi: 10.1093/icb/icr097 21841184

[48] Verberk WCEP, Atkinson D, Hoefnagel KN, Hirst AG, Horne CR, Siepel H. Shrinking body sizes in response to warming: explanations for the temperature–size rule with special emphasis on the role of oxygen. Biological Reviews. 2021;96(1):247–68. doi: 10.1111/brv.12653 32959989 PMC7821163

[49] Jutfelt F, Norin T, Åsheim ER, Rowsey LE, Andreassen AH, Morgan R, et al. ‘Aerobic scope protection’reduces ectotherm growth under warming. Funct Ecol. 2021;35(7):1397–407.

[50] Cook AM. Growth and survival of age 0+ Shubenacadie River striped bass (Morone saxatilis) in relation to temperature and salinity [MSc Dissertation]: Nova Scotia Agricultural College; 2003.

[51] Cox DK, Coutant CC. Growth dynamics of juvenile striped bass as functions of temperature and ration. Trans Am Fish Soc. 1981;110(2):226–38. doi: 10.1577/1548-8659(1981)110&lt;226:GDOJSB&gt;2.0.CO;2

[52] Duston J, Astatkie T, MacIsaac P. Effect of body size on growth and food conversion of juvenile striped bass reared at 16–28 C in freshwater and seawater. Aquaculture. 2004;234(1–4):589–600.

[53] Otwell WS, Merriner JV. Survival and growth of juvenile striped bass, Morone saxatilis, in a factorial experiment with temperature, salinity and age. Trans Am Fish Soc. 1975;104(3):560–6. doi: 10.1577/1548-8659(1975)104&lt;560:SAGOJS&gt;2.0.CO;2

[54] Beemelmanns A, Ribas L, Anastasiadi D, Moraleda-Prados J, Zanuzzo FS, Rise ML, et al. DNA methylation dynamics in Atlantic salmon (Salmo salar) challenged with high temperature and moderate hypoxia. Frontiers in Marine Science. 2021;7:604878.

[55] Navarro-Martín L, Martyniuk CJ, Mennigen JA. Comparative epigenetics in animal physiology: An emerging frontier. Comparative Biochemistry and Physiology Part D: Genomics and Proteomics. 2020;36:100745. doi: 10.1016/j.cbd.2020.100745 33126028

[56] Venney CJ, Wellband KW, Normandeau E, Houle C, Garant D, Audet C, et al. Thermal regime during parental sexual maturation, but not during offspring rearing, modulates DNA methylation in brook charr (Salvelinus fontinalis). Proceedings of the Royal Society B. 2022;289(1974):20220670. doi: 10.1098/rspb.2022.0670 35506232 PMC9065957

[57] Penny FM, Bugg WS, Kieffer JD, Jeffries KM, Pavey SA. Atlantic sturgeon and shortnose sturgeon exhibit highly divergent transcriptomic responses to acute heat stress. Comparative Biochemistry and Physiology Part D: Genomics and Proteomics. 2023;45:101058. doi: 10.1016/j.cbd.2023.101058 36657229

[58] Gans I, Hartig EI, Zhu S, Tilden AR, Hutchins LN, Maki NJ, et al. Klf9 is a key feedforward regulator of the transcriptomic response to glucocorticoid receptor activity. Scientific reports. 2020;10(1):1–16. 32651405 10.1038/s41598-020-68040-z PMC7351738

[59] Yu Y, Li C, Wang Y, Wang Q, Wang S, Wei S, et al. Molecular cloning and characterization of grouper Krϋppel-like factor 9 gene: Involvement in the fish immune response to viral infection. Fish Shellfish Immunol. 2019;89:677–86. 30905839 10.1016/j.fsi.2019.03.041

